# Preparation of Alumina Ceramics via a Two-Step Sintering Process

**DOI:** 10.3390/ma18081789

**Published:** 2025-04-14

**Authors:** Jiawen Yang, Liwen Lei, Jinyong Zhang

**Affiliations:** 1 State Key Laboratory of Advanced Technology for Materials Synthesis and Processing, Wuhan University of Technology, Wuhan 430070, China; yjw18571343863@163.com (J.Y.);; 2 Hubei Longzhong Laboratory, Xiangyang 441000, China

**Keywords:** spark plasma sintering, pressureless sintering, two-step sintering, alumina ceramics, apparent activation energy

## Abstract

This paper investigates grain growth and densification kinetics in alumina ceramics subjected to spark plasma sintering (SPS) and conventional pressureless sintering (CS). The findings reveal that, under both sintering conditions, grain growth primarily occurs after reaching the sintering ‘freezing point’. The analysis of densification kinetics indicates that the activation energies of densification of alumina ceramics are 173.6 KJ/mol and 261.2 KJ/mol during the early stage, and 362.2 KJ/mol and 383.7 KJ/mol in the late stage for SPS and CS conditions, respectively. Therefore, a two-step sintering method (TSS) is proposed, where SPS rapidly sinters alumina powder to reach the grain growth ‘freezing point’, and then, the sintered bodies are subjected to CS to obtain dense alumina ceramics. The results show that the flexural strength of alumina prepared using this TSS can reach 489.6 MPa, about 19% improvement over those processed solely through CS.

## 1. Introduction

Alumina ceramics are among the most widely used structural ceramics, renowned for their exceptional strength and hardness and good chemical and thermal stability [1,2,3]. Conventionally, high-temperature and high-pressure sintering techniques such as hot pressing (HP) or hot isostatic pressing (HIP) are needed to achieve alumina ceramics with fully dense microstructures, ensuring excellent mechanical performances [4,5,6]. However, these high-temperature, high-pressure sintering processes demand costly equipment and often result in low sintering efficiencies (limited sintering intervals), significantly raising the cost of product fabrication. To reduce the cost of high-performance ceramics, researchers have explored alternatives to the expensive pressure sintering methods by opting for the common pressureless sintering (CS) process. This approach aims to eliminate the limitation of hot-pressure sintering equipment and improve yield in a single sintering process. However, CS often leads to excessive grain growth in the sintered samples, making it challenging to achieve higher-density materials that can match the performance of those produced by HP [7,8,9].

Two-step sintering (TSS) was first proposed by Chen et al. [10] in 2000. A rapid densification process was utilized to obtain green bodies with a relative density of about 85%, which a prolonged low-temperature CS followed to achieve dense nanocrystalline alumina. Chen et al. [10] concluded that the amount of material diffusion required to achieve densification during CS can be significantly reduced by increasing the density of the sintered green bodies, thus suppressing grain growth due to diffusion. The results of their work even indicated that the final densification of pre-sintered ceramics can be achieved entirely by surface diffusion, which does not lead to grain growth. This work has provided new ideas for innovation in the sintering process of high-performance ceramics.

Several researchers have explored this topic, following the work of Chen et al. Filipe et al. [11] conducted a systematic study on the process optimization of TSS. The results revealed that employing a peak temperature (T_1_) to enhance the relative density of the green bodies, followed by an extended low-temperature isothermal stage (T_2_) and optimizing the cooling rate between T_1_ and T_2_, can also achieve the sintering of nanoscale dense ZrO_2_ ceramics.

On the other hand, Kim et al. [12] found that using this method of an initial relatively low temperature followed by a high temperature can prepare transparent alumina ceramics. The results showed that the relatively low temperature could homogenize the grains of the sintered powders, thus inhibiting and homogenizing the grain growth in the final high-temperature sintering stage. Moreover, Zhu et al. [13] fabricated Nd-doped YAG ceramics with greatly enhanced optical properties based on the method of Kim et al. and found that the samples had a non-porous and homogeneous microstructure with a transparency of up to 84.98% in the visible and near-infrared bands and 87% in the mid-infrared band.

However, there is an obvious limitation in the practical application of these works. The second step in TTS of Chen et al. [10] mainly focused on the refinement and homogenization of the ceramic grains and the performance enhancement without considering the economic cost. It, therefore, adopted a CS process of more than 10 h. Such a long sintering cycle greatly affects the economy of high-performance ceramics. Whether a relatively optimal balance between performance enhancement and economy can be reached, thus promoting the development of related technical ceramics engineering, is a significant question to explore. For this reason, with alumina as the material object in this study, a new sintering strategy is proposed as an alternative to the expensive HP process by investigating its densification kinetics and grain growth kinetics during spark plasma sintering (SPS) and CS.

## 2. Materials and Methods

The alumina powder used in the experiments was grade AKP-50 from Sumitomo Chemical, Japan, model AKP-50, with an average particle size of 220 nm and a mass purity of 99.9%.

To achieve a high heating rate, pressure sintering was performed using an SPS sintering furnace (Shanghai Chenhua Technology Co., Ltd., Shanghai, China), where alumina raw material powder was loaded into a graphite mold with an inner diameter of 30 mm and a conductive ceramic boat, and preloaded with a sintering pressure of 30 MPa. The height of the billet was controlled to approximately 10 mm by adjusting the amount of material added. CS was conducted in a modified direct resistance heating furnace (Shanghai Chenhua Technology Co., Ltd., Shanghai, China). Sheet blanks of φ30 mm × 10 mm, pre-pressurized by 150 MPa cold isostatic pressure, were placed between two Mo resistors heated up and down simultaneously. The temperatures during sintering were tested with the same type of infrared temperature detector and were cross-calibrated.

Heating regime: The sample was first heated rapidly to 600 °C to start temperature measurement and control of heating. The samples were heated to a set sintering temperature (1700 °C for CS and 1500 °C for SPS) at a fixed ramp-up rate (5 °C/min, 50 °C/min, and 150 °C/min). Subsequently, the sintering was terminated and cooled down naturally.

Neither of the two sintering furnaces used an insulating layer, allowing both to cool to about 600 °C rapidly within about 5 min. It was generally assumed that the diffusion of alumina ceramics within 600 °C and the creep at 30 MPa pressure were both small and negligible. This assumption was supported by the relatively small change in the densification of the samples during the actual sintering process as the temperature was ramped up to 600 °C, as detailed in the experimental results section of the main text.

During the SPS process, the displacement curve of the mold indenter was recorded using a displacement meter. Based on the principle of constant mass and assuming no mold deformation during the sintering process, the relative density of the samples can be calculated over time. Our comparative study, which involved a thicker mold jacket and a higher-strength graphite material, indicated that the dimensional changes of the mold during the sintering process were minimal, resulting in an error in the sintering densification calculation of less than 0.5%. The theoretical density used for calculating relative density was 3.98 g/cm^3^.

In this study, a two-step sintering process (TSS) was employed. Initially, spark plasma sintering (SPS) was used to sinter alumina bodies to their “freezing point” rapidly. Subsequently, conventional pressureless sintering (CS) was used to achieve full densification, resulting in highly dense and mechanically robust sintered bodies. The specific process was as follows: Initially, the sample was rapidly heated to T_1_ (1300 °C, holding time 5 min) using SPS at a consistent pressure. After cooling, the sample was removed and placed in a pressureless sintering furnace for the second step of CS. The CS adopted a rapid heating process (the heating rate is 20 °C/min) and the sintering temperature T_2_ was 1450 °C with a holding time of 2 h. For comparison, CS was used to obtain dense alumina ceramics with a sintering temperature of 1500 °C and a holding time of 2 h.

The sintered samples were smoothed and polished, and the density of the sintered alumina bodies was determined using the Archimedes drainage method, facilitating the calculation of the relative density of the final. The results showed that the calculated relative density of the final sample obtained was within 1% error margin compared to the relative density obtained through indenter displacement, validating the accuracy of the aforementioned method. The microstructure of the samples, obtained under different conditions, was analyzed using scanning electron microscopy (JSM-IT800, JEOL Ltd., Tokyo, Japan). Before observation, the samples underwent a progressive abrasion using diamond polishing disks, finishing with a 4000-mesh abrasive. Subsequently, they were subjected to high-temperature etching in a muffle furnace at 1000 °C for 60 min. Flexural strength was measured using a universal testing machine (CMT6503, MTS Systems (China) Co., Ltd. Shenzhen, China), using a three-point flexural method with a span of 15 mm and a loading rate of 0.5 mm/min, with specimens measuring 2 mm × 3 mm × 18 mm. The hardness test was carried out using a Vickers hardness tester (Tukon2500B, Buehler Ltd., Lake Bluff, Illinois, USA., with a specimen size of 2 mm × 3 mm × 10 mm, with a load of 9.8 N for 15 s.

## 3. Results and Discussion

**Figure 1:**
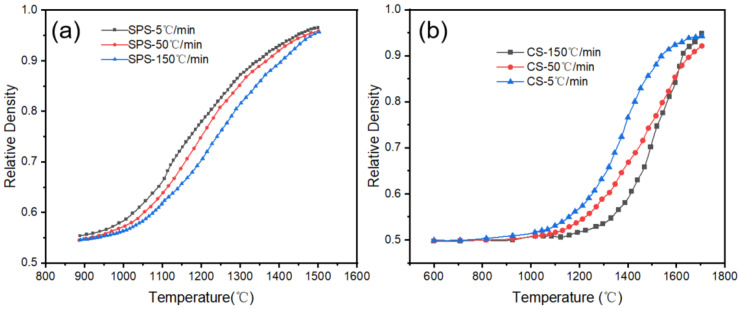
Variation in relative density of sintered alumina bodies with temperature at different constant heating rates. (**a**)SPS, (**b**) CS.

The variation in densification rate with temperature during SPS and CS of alumina at different heating rates is illustrated in Figure 1. Due to the constant heating mode used in the sintering process, there exists a linear relationship between temperature and sintering time; consequently, the changes in density over time correspond with the trends in density relative to temperature. As illustrated in Figure 1, the final densities of the samples produced via SPS exceeded 94%. Due to the high temperature in CS, the final densities of the samples also reached high levels, with increasing heating rates; the final densities of the samples were 94.2%, 92.1%, and 94.8%, respectively. It is evident that all samples have reached the final sintering stage and have essentially completed the sintering process.

The rate of temperature increase affects both CS and SPS to some extent, especially when the temperature increase reaches 150 °C/min. At this point, the rate of densification and the final densification of the sample in CS were enhanced. This enhancement occurs primarily because the rapid heating rate suppresses the ineffective grain growth caused by the material diffusion during the heating process while simultaneously leading to incomplete surface diffusion. The surface diffusion mechanism, which typically dominates at lower temperatures, does not proceed sufficiently and becomes prominent within the high-temperature sintering zone. This mechanism competes with conventional volume diffusion, which usually prevails at high temperatures. This competition enhances the role of particle rearrangement in the densification process [14]. Similar results were reported by Zou et al. [15]. This effect is not apparent when the heating rate is within 50 °C/min. In the SPS process, the inhibitory effect of rapid heating on grain growth is significantly mitigated due to the presence of an applied pressure that can promote grain rearrangement in the early sintering stage.

**Figure 2:**
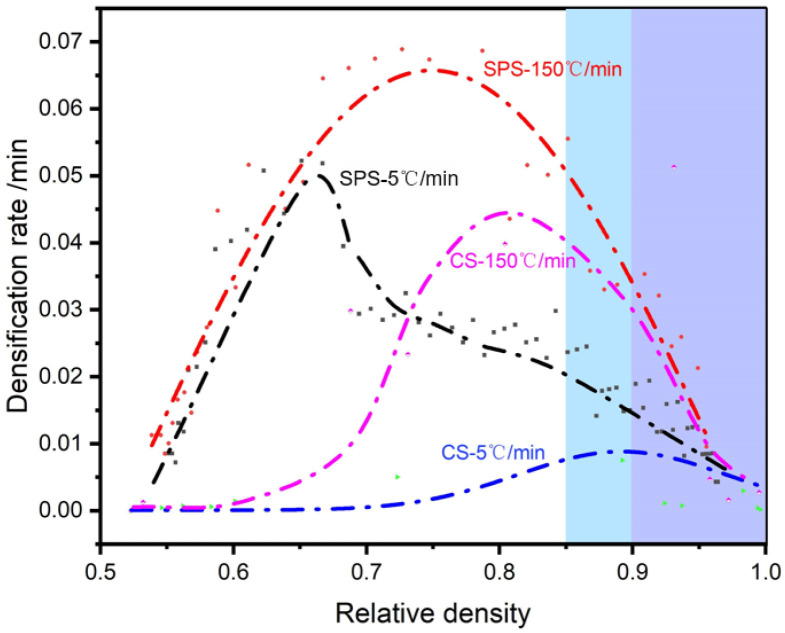
Variation in sintering densification rate with relative densities of alumina samples under typical process conditions.

To further illustrate the influence of the process on the densification behavior during sintering, Figure 2 presents the variation in the sintering densification rate with the densification of the sample sintered under typical sintering conditions. As demonstrated in Figure 2, the densification rate first increases and then experiences a decline as the densification of the samples progresses. Furthermore, where the densification rate increases more rapidly, its following decline will be faster. And all the densification rates finally reach almost the same small level, under 0.01/min.

The two stages of sintering can be described as the pre-sintering and post-sintering stages according to the treatises of Coble [16] and Kang [17]. In the pre-sintering powder particles in the pressure or surface or grain boundary diffusion under rapid convergence, the inter-particle pores gradually evolved into closed pores, sintering into the final stage. In the traditional pressureless sintering theory, surface and grain boundary diffusion is generally believed to dominate the densification process in the pre-sintering stage. In contrast, when the pores are closed in the final stage of sintering, the densification is mainly realized by body diffusion. Since the body diffusion coefficient is much smaller than the surface diffusion, and the corresponding activation is much higher, the densification rate decreases significantly in the final sintering stage. However, the macroscopic division between pre-sintering and post-sintering and the effect of the pressure remain incompletely understood. Previous studies showed that these factors are intertwined with the intrinsic properties of the powder, initial particle size, and other variables. For the demarcation point between pre-sintering and post-sintering, Chen et al. [10] proposed that it occurs at around 85% relative density, referring to it as the sintering “freezing point”. This point is regarded as a critical process control parameter in the two-step sintering method. Figure 2 reveals that at the “freezing point” of about 90%, the densification rate (first-order derivative of the rate) decreases rapidly, and the difference between SPS and CS almost disappears at the same heating regime. Additionally, the effect of an excessively high heating rate on densification is evident in Figure 2, aligning with the previous analysis. This phenomenon has also been corroborated by recent advancements in the flash sintering (FS) process [18].

**Figure 3:**
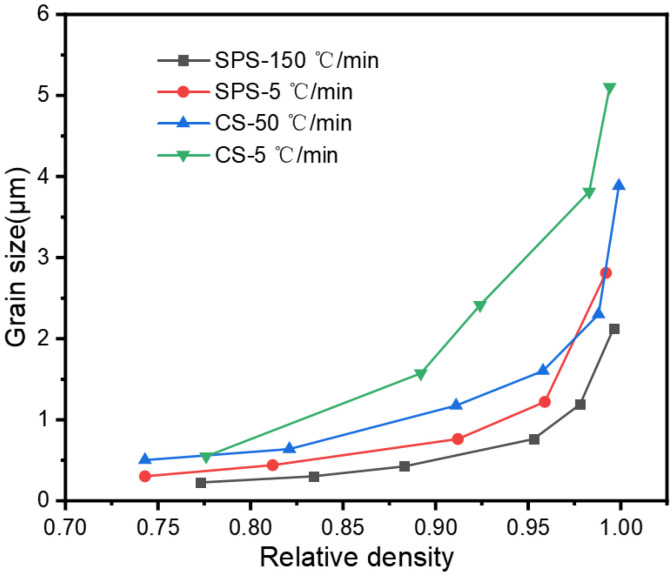
Plot of relative density versus grain size in SPS and CS.

Grain size is a critical factor affecting the properties of sintered bodies, and this study also examined the grain growth characteristics during the sintering process. Figure 3 illustrates the relationship between the relative density of the samples versus grain size for the SPS and CS sintering processes. The alumina grains within the sintered bodies grew from an initial average of 220 nm to 5.1 μm, 3.8 μm, 2.8 μm, and 2.1 μm in the sintering condition of CS-5 °C/min, CS-50 °C/min, SPS-5 °C/min, and SPS-150 °C/min, respectively. We can see that whether it is SPS sintering or CS sintering, the grain size growth is relatively slow before the density reaches 90%, and when the relative density reaches 90%, the grain size has a rapid growth. For both SPS sintering and CS sintering, increasing the heating rate can inhibit the growth of grains, because rapid heating can inhibit the ineffective growth of grains caused by material diffusion during the heating process, which is consistent with what we mentioned earlier. This result aligns with the report of Guillaume et al. [19].

SPS can enhance the densification rate of sintering and inhibit the growth of grains before reaching the “freezing point”. However, after this point, the level of promotion and inhibition significantly reduced, eventually converging to the pressureless sintering. This contrasts with the study by Chen et al. [10], primarily because of the cooling strategy of TSS by Chen et al. during the final stage of sintering, but the conventional high-temperature sintering was still used.

To further elucidate the differences between SPS and CS, the different sintering mechanisms mentioned above were also analyzed using the CHR method [20]. The densification rate versus sintering apparent activation energy relationship in the CHR method can be expressed as follows [20]:

(1) $$\frac{d\rho }{dt}=A\frac{e^{-Q/RT}}{T}\frac{f(\rho )}{G^{n}}$$

where A is a material constant, R the gas constant, f (ρ) is a density-only dependent function, G is the grain size, n is a grain size index dependent on the dominant diffusion mechanism (n = 3 for lattice diffusion, and n = 4 for grain boundary diffusion), and Q is the apparent activation energy reflecting the kinetic mechanism of sintering.

Taking logarithms on both sides of Equation (1), this can be transformed into the following:

(2) $$ln(T\frac{d\rho }{dt})=-\frac{Q}{RT}+ln[f(\rho )]+lnA-nlnG$$

ln [T*(dρ/dt)] is linearly related to 1/T, and the corresponding slope is −Q/R. When the heating rate does not significantly affect the densification process, by substituting the results of the previous experimental process of the relative density at different heating rates and the change in grain size with time and temperature during the sintering process, the apparent activation energies at different relative densities (to varying stages of the sintering densification process) can be calculated under different process conditions. The apparent activation energy can be calculated for different relative densities under different process conditions (different stages of the sintering densification process).

**Figure 4:**
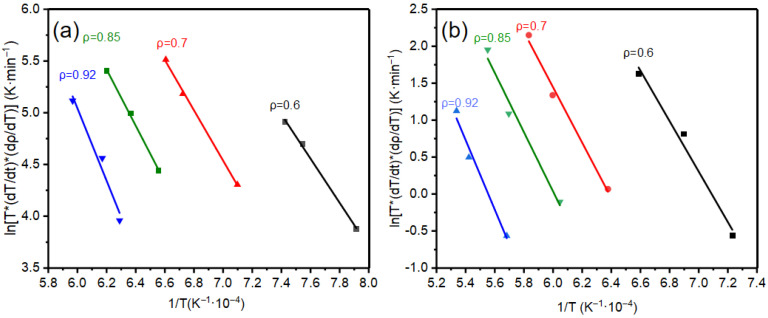
Arrhenius plots of ln [T *(dρ/dt)] vs. 1/T for non-isothermal sintering at the same relative density; (**a**) SPS Arrhenius plot, (**b**) CS Arrhenius plot.

The results of the kinetic analysis of the densification process for SPS and CS alumina by the CHR method are presented in Figure 4. Figure 4 reveals that the slope of the ln [T*(dρ/dt)] vs. 1/T straight line is gradually increasing with the rise in the relative density, regardless of whether it pertains to SPS or CS. The magnitude of this variation remains relatively small in a specific interval, which aligns with findings reported in the literature [15].

Figure 4 illustrates the relationship between the apparent activation energy and relative density. The figure revealed that the apparent activation energy Q of sintering densification in the SPS process rises from 173.6 kJ/mol at 60%, 197.8 kJ/mol at 70%, and quickly rises to 362.2 kJ/mol at 92%; Q in the CS process rises from 261.3 kJ/mol at 60%, 278.3 kJ/mol at 70%, and finally reaches 383.7 kJ/mol at 92%. In addition, all R^2^ values are greater than or equal to 96.9, so we believe that the linear fitting data are reliable.

For CS alumina, various studies have reported differing results, He et al. [21] calculated the sintering activation energy of about 342 KJ/mol (1600 °C, 1650 °C, 1700 °C) for an initial particle size of 700 nm by constant temperature experiments. Meanwhile, Fang et al. [22] reported that the sintering activation energies for powders with average particle sizes of 240 nm and 210 nm were 420 KJ/mol and 452 KJ/mol, respectively. These discrepancies may stem from the differing types of powders the researchers utilize and the varying experimental conditions.

**Figure 5:**
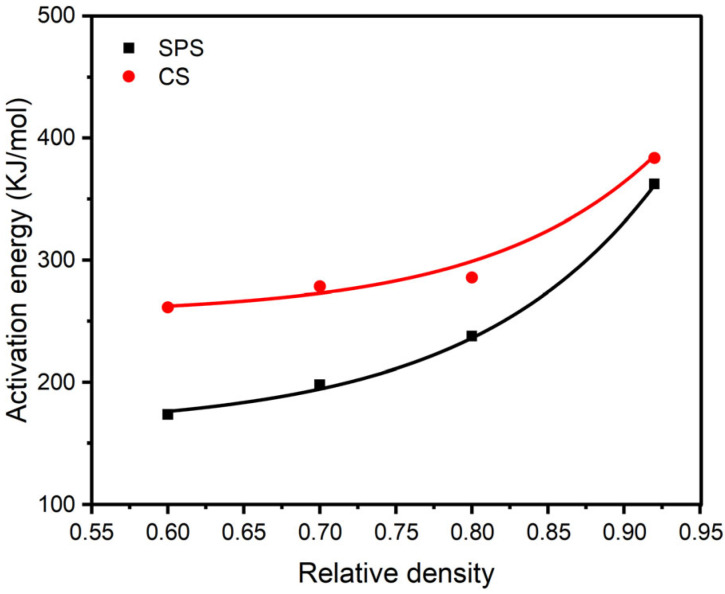
Plot of the difference between the apparent activation energy of SPS and CS.

From Figure 5, it is evident that the disparity between the activation energies for SPS and CS densification decreases significantly as the relative density of the samples increases, ultimately converging at about 92%. This observation indicates that the densification patterns during the late sintering stage tend to be the same or similar in both cases. In conjunction with the aforementioned grain growth law, this study proposes a new sintering strategy, using SPS to rapidly obtain green bodies whose relative density reaches the “freezing point”, and then sintering it with CS at a rapid temperature rise to obtain short-cycle, high-performance ceramics.

Table 1 shows the experimental scheme of the two-step sintering method (TSS, T_1_ = 1300 °C, holding time 5 min, T_2_ = 1450 °C, holding time 2 h) and traditional pressureless sintering (CS, 1500 °C, holding time 2 h).

**Figure 6:**
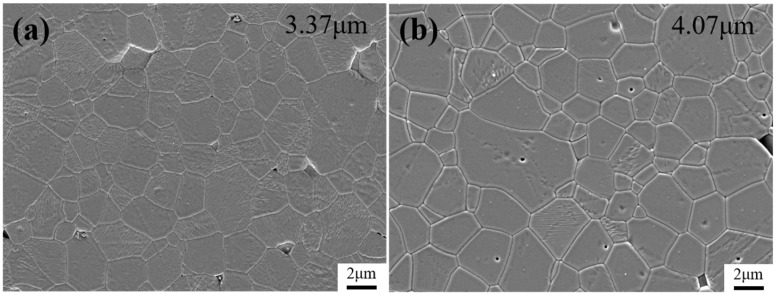
Etched SEM image of sintered alumina bodies after polishing. (**a**) TSS (**b**) CS.

**Figure 7:**
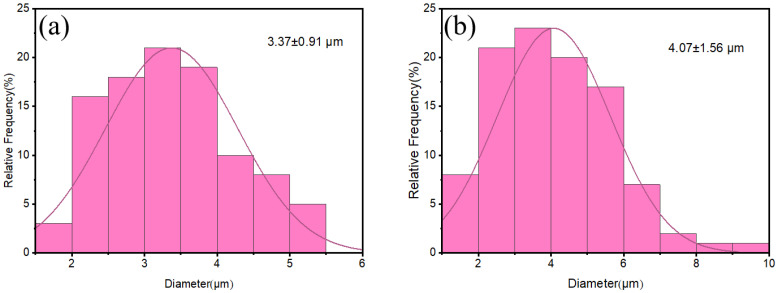
Grain distribution of sintered alumina body. (**a**) TSS (**b**) CS.

Figure 6 presents SEM images of the polished and etched sintered alumina bodies produced by the two-step sintering method. It can be seen from the figure that the alumina ceramics prepared by the two sintering processes reached densification (at this time, the relative densities of the alumina obtained by the TSS and the CS reached 98.8% and 98.6%, respectively). Figure 7 shows the grain distribution of TSS- and CS-sintered alumina. The average grain size of the sintered alumina bodies obtained by the TSS is 3.37 ± 0.91 μm. The average grain size of the conventional CS alumina ceramics using the same powder is about 4.07 ± 1.56 μm. Compared with the conventional CS alumina ceramics using the same powder, the present TSS reduces average grain size by about 17.2% while achieving the same densities. The strategy proposed in this work effectively reduces the sintering temperature required for final sintering and successfully inhibits grain growth.

**Figure 8:**
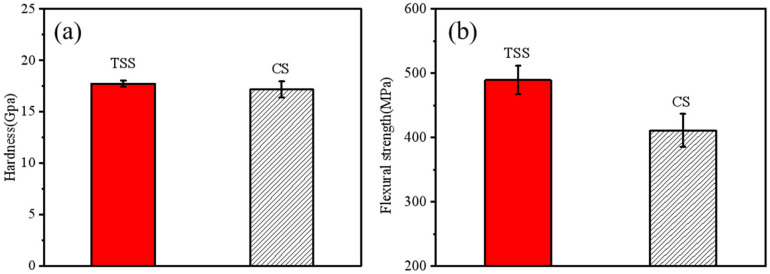
Mechanical properties of alumina ceramics: (**a**) hardness, (**b**) flexural strength.

Typical mechanical properties of sintered alumina bodies prepared using TSS and CS methods are presented in Figure 8. The hardness and flexural strength of the TSS samples were 17.7 GPa and 489.6 MPa, respectively. In contrast, the hardness and flexural strength of the sintered alumina bodies obtained by the CS method were 17.1 GPa and 411.4 MPa, respectively. It was observed that the hardness of the two sintered bodies was comparable. The Hall–Petch effect [23] describes the relationship between the hardness of ceramic materials and grain size and porosity. Previous research has indicated that the effect of grain size becomes negligible when the grain size exceeds 0.25 μm [24]. In this study, all sample grain sizes were larger than 3 μm, confirming that the effect of grain size on hardness was negligible in this context, consistent with findings reported by Liu et al. [25]. The 19% enhancement in the flexural strength of the samples obtained using the TSS method compared to those made using the CS method can be attributed to the reduction in grain size [25,26].

## 4. Conclusions

This paper analyzes and compares the growth and densification kinetics of SPS and CS grains. It is concluded that the growth of grains mainly occurs after the sintering “freezing point” for both SPS and CS. The activation energy of densification in the initial stage of SPS is lower than that of CS. In contrast, the densification pattern tends to be the same or similar in the final stages of sintering. A new two-step sintering (TSS) strategy is proposed, which involves rapid hot pressing with SPS to achieve green bodies with relative densities up to the “freezing point”, followed by fast-temperature CS sintering to produce short-cycle, high-performance ceramics. Alumina ceramics were prepared using TSS approach (T_1_ = 1300 °C, holding time 5 min, T_2_ = 1450 °C holding time 2 h) and were compared with alumina prepared using CS (1500 °C, 2 h holding time). The results demonstrated that the flexural strength of alumina prepared by the TSS method reached 489.6 MPa, representing an improvement of about 19% compared to the alumina obtained through CS for dense ceramic production. The effective TSS approach also reduces the final temperature required for CS, leading to smaller grain sizes.

## Figures and Tables

**Table 1:** Experimental scheme.

Sample	T_1_ (°C)	Relative Density of Initial Blank (%)	T_2_ (°C)	Relative Density (%)
TSS	1300	95.4	1450	98.8
CS	1500	——	——	98.6

## Data Availability

The data presented in this study are available on request from the corresponding author.
